# The causal effects between gut microbiota and hemorrhagic stroke: a bidirectional two-sample Mendelian randomization study

**DOI:** 10.3389/fmicb.2023.1290909

**Published:** 2023-12-22

**Authors:** Yingjie Shen, Hao Liu, Xiangyi Meng, Aili Gao, Yansong Liu, Wei Ma, Hongsheng Liang, Fulan Hu

**Affiliations:** ^1^Department of Neurosurgery, The First Affiliated Hospital of Harbin Medical University, Harbin, China; ^2^Clinical Laboratory of Molecular Biology, The First Affiliated Hospital of Harbin Medical University, Harbin, China; ^3^School of Life Science, Northeast Agricultural University, Harbin, China; ^4^NHC Key Laboratory of Cell Transplantation, Department of Neurosurgery, The First Affiliated Hospital of Harbin Medical University, Harbin, China; ^5^Department of Biostatistics and Epidemiology, School of Public Health, Shenzhen University Medical School, Shenzhen, China

**Keywords:** causal inference, gut microbiota, hemorrhagic stroke, Mendelian randomization, odds ratio

## Abstract

**Background:**

Recent studies have suggested that the composition of gut microbiota (GM) may change after intracerebral hemorrhage. However, the causal inference of GM and hemorrhagic stroke is unknown. Mendelian Randomization (MR) is an effective research method that removes confounding factors and investigates the causal relationship between exposure and outcome. This study intends to explore the causal relationship between GM and hemorrhagic stroke with the help of MR.

**Methods:**

Univariable and multivariable MR analyses were performed using summary statistics of the GM (*n* = 18,340) in the MiBioGen consortium vs. the FinnGen consortium R9 summary statistics (intracerebral hemorrhage, subarachnoid hemorrhage, and nontraumatic intracranial hemorrhage). Causal associations between gut microbiota and hemorrhagic stroke were analyzed using inverse variance weighted, MR-Egger regression, weighted median, weighted mode, simple mode, and MR-PRESSO. Cochran’s *Q* statistic, MR-Egger regression, and leave-one-out analysis were used to test for multiplicity and heterogeneity of instrumental variables. Separate reverse MR analyses were performed for microbiota found to be causally associated with hemorrhagic stroke in the forward MR analysis. Also, multivariate MR analyses were conducted after incorporating common confounders.

**Results:**

Based on the results of univariable and multivariate MR analyses, *Actinobacteria (phylum)* (OR, 0.80; 95%CI, 0.66–0.97; *p* = 0.025) had a protective effect against hemorrhagic stroke, while *Rikenellaceae RC9 gut group (genus)* (OR, 0.81; 95%CI, 0.67–0.99; *p* = 0.039) had a potential protective effect. Furthermore, *Dorea (genus)* (OR, 1.77; 95%CI, 1.27–2.46; *p* = 0.001), *Eisenbergiella (genus)* (OR, 1.24; 95%CI, 1.05–1.48; *p* = 0.013) and *Lachnospiraceae UCG008 (genus)* (OR, 1.28; 95%CI, 1.01–1.62; *p* = 0.041) acted as potential risk factors for hemorrhagic stroke. The abundance of *Dorea (genus)* (β, 0.05; 95%CI, 0.002 ~ 0.101; *p* = 0.041) may increase, and that of *Eisenbergiella (genus)* (β, −0.072; 95%CI, −0.137 ~ −0.007; *p* = 0.030) decreased after hemorrhagic stroke according to the results of reverse MR analysis. No significant pleiotropy or heterogeneity was detected in any of the MR analyses.

**Conclusion:**

There is a significant causal relationship between GM and hemorrhagic stroke. The prevention, monitoring, and treatment of hemorrhagic stroke through GM represent a promising avenue and contribute to a deeper understanding of the mechanisms underlying hemorrhagic stroke.

## 1 Introduction

Hemorrhagic stroke is a sudden condition caused by the rupture of a specific blood vessel in the brain (Di Biase et al., 2023). It is classified based on two main types of bleeding sites: intracerebral hemorrhage (ICH) within the brain parenchyma and subarachnoid hemorrhage (SAH) within the subarachnoid space (Ikram et al., 2012; Hoh et al., 2023). In the year 2019, the global burden of stroke exhibited notable figures: 12.2 million incident cases (95% UI 11.0–13.6), 101 million prevalent cases (95% UI 93.2–111), 143 million disability-adjusted life-years attributed to stroke (95% UI 133–153), and 6.55 million deaths stemming from this condition (95% UI 6.00–7.02). Within this spectrum, intracerebral hemorrhage accounted for 27.9% of all incident strokes, while subarachnoid hemorrhage constituted 9.7% (Collaborators, 2021). Such statistics shed light on the significant global health impact of stroke in terms of morbidity and mortality, necessitating sustained efforts in research and intervention to mitigate its consequences. The hemorrhagic stroke seriously affects the prognosis of the patients and, at the same time, creates a heavy financial burden for the patients. Therefore, early identification of risk factors for hemorrhagic stroke is necessary. So far, hypertension, diabetes mellitus, alcohol, obesity, smoking, and genetic factors are risk factors for hemorrhagic stroke, and the irrational development of these factors promotes cerebrovascular instability and thus contributes to the occurrence of hemorrhagic events (Magid-Bernstein et al., 2022). In addition, the link between dietary and intestinal factors and nerves is gradually being emphasized, although there is not sufficient evidence yet (Li et al., 2018).

The human gut is characterized by a microbiota in which bacteria are the dominant species (Lozupone et al., 2012). Recently, there has been increasing evidence that the gut microbiota (GM) is closely related to host health and is involved in the development of the etiology of a wide range of human diseases (Clemente et al., 2012). GM may communicate with the central nervous system through the microbiota-gut-brain (MGB) axis, including immune responses, neural connections, and so on (Cryan et al., 2019). Recent studies have demonstrated that gut microbial alterations after cerebral hemorrhage are a key factor in promoting secondary brain injury and prognosis (Zou et al., 2022). In addition, several studies have demonstrated that dysregulation of GM and their metabolites may have an impact on a variety of phenotypes, including vascular inflammation, blood pressure, lipid metabolism, and tissue injury and repair, which may suggest that GM may indirectly contribute to the development of cerebrovascular disease (Witkowski et al., 2020). However, GM is a large class of populations, and it is unclear whether one or more bacterial traits are involved in the development of hemorrhagic stroke. Potential shortcomings of clinical studies and preclinical trials, such as limited sample size and retrospective design, have hindered our determination of a causal relationship between GM and hemorrhagic stroke.

Mendelian randomization (MR) is a technique that employs single nucleotide polymorphisms (SNPs) as instrumental variables (IVs) obtained from a genome-wide association study (GWAS) to ascertain the causal relationship between an exposure and an outcome (Emdin et al., 2017). By Mendel’s principles of inheritance, at conception, alleles are transmitted randomly from parent to child, akin to the random allocation principle in randomized controlled trials. Therefore, MR can circumvent the limitations found in observational studies, such as prejudice and reverse causation, and enable the inference of causal relationships between complex diseases (Bowden and Holmes, 2019). MR has been widely used to explore the relationship between GM and a variety of disease phenotypes, such as immunologic disorders (Xu et al., 2021), psychiatric disorders (Ni et al., 2021), and cardiovascular diseases (Meng et al., 2023). In this study, we will discuss the causal effect of GM on hemorrhagic stroke and the corresponding inverse relationship using MR analysis based on GWAS summary statistics from the MiBioGen and FinnGen consortiums.

## 2 Materials and methods

### 2.1 Ethics statement and study design

This study did not require additional ethical approval since we used publicly available data, which had already received approval from the appropriate ethical and institutional review boards. We selected SNPs significantly associated with exposure as IVs based on strict inclusion and exclusion criteria and included relevant IVs for MR analysis and sensitivity analyses through hypothetical criteria for MR.

**Figure 1:**
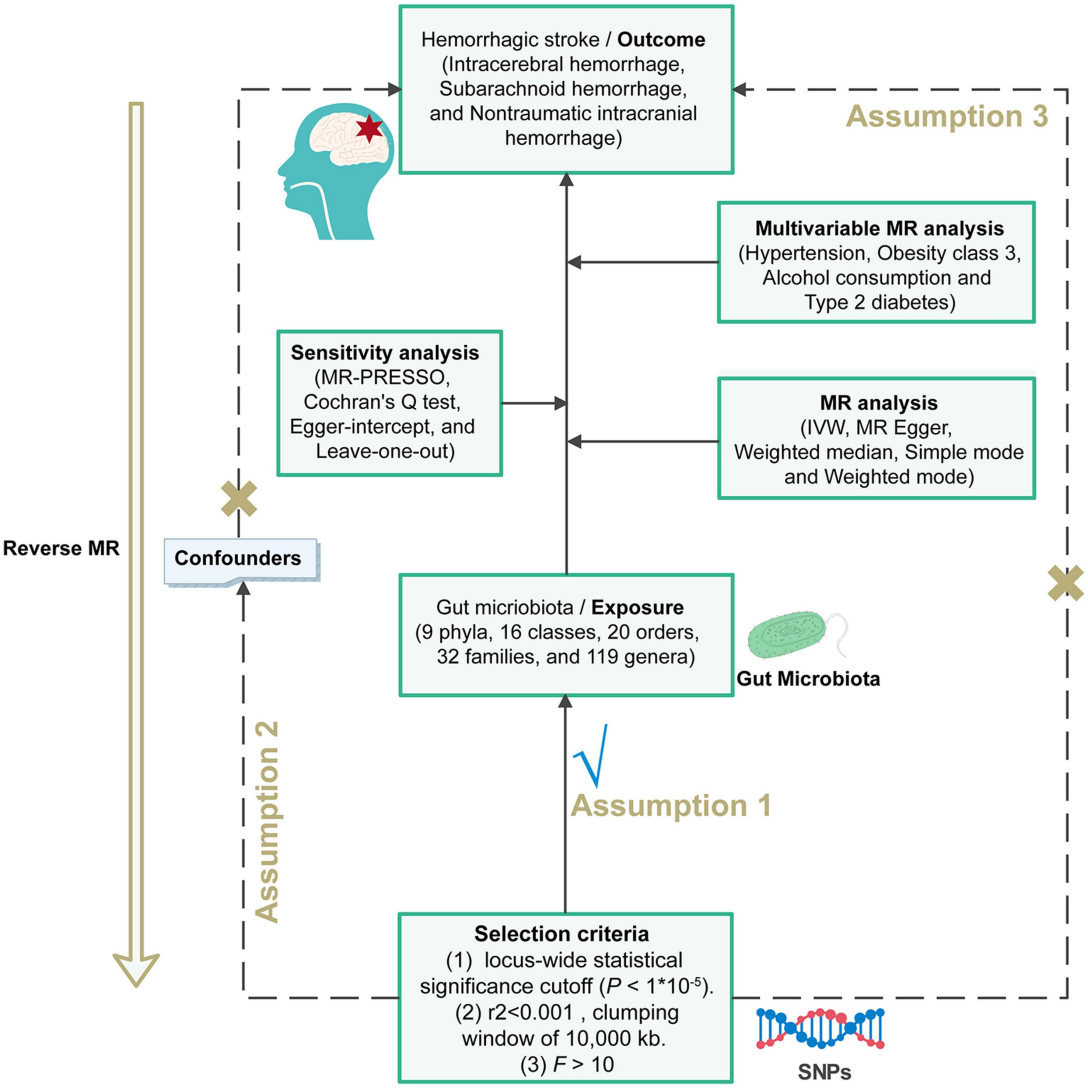
Flowchart of the MR randomization study and key assumptions. Three key assumptions for a valid Mendelian randomization analysis. (I) the instrumental variable selected from the dataset was significantly associated with exposure; (II) the instrumental variable was not associated with any unknown confounders of exposure; and (III) the instrumental variable could only be associated with the outcome through exposure. MR, Mendelian randomization; SNPs, single nucleotide polymorphisms; IVW, inverse-variance weighted; MR-PRESSO, MR pleiotropy residual sum and outlier.

We used GM and hemorrhagic stroke as exposures to observe their effects on outcomes separately. All MR analysis of this study was executed under three basic assumptions (Davies et al., 2018). Firstly, we need to ensure that genetic variants are independently and significantly associated with the exposure, a requirement that we fulfilled by utilizing *p*-values and *F*-statistics from correlation tests. Secondly, we must establish that there is no association between the genetic variants related to the exposure and the outcomes. Lastly, the third premise assumes these variants should not correlate with any confounding variables (Figure 1).

This study is reported in accordance with the Strengthening the Reporting of Observational Studies in Epidemiology Using Mendelian Randomization guidelines (STROBE-MR) (Skrivankova et al., 2021) (Supplementary Table S1).

### 2.2 Data source

We acquired the most recent GWAS dataset of the human gut microbiome from the MiBioGen consortium. This dataset is the largest, multi-ethnic, genome-wide analysis of human autosomal genetic variation and the gut microbiome, specifically designed to investigate host-genetics-microbiome associations (Wang et al., 2018; Kurilshikov et al., 2021). The study involved 18,340 participants from diverse countries and regions, predominantly representing European groups. The consortium utilized standardized analytical pipelines for both microbiota phenotype and genotype, ensuring uniform data processing methods. As a result of this comprehensive study, we successfully identified 211 bacterial taxa, including 9 *phyla*, 16 *classes*, 20 *orders*, 35 *families* (of which 3 were unknown), and 131 *genera* (of which 12 were unknown) (Kurilshikov et al., 2021). For our study, we excluded 15 microbial taxa with missing information regarding *families* and *genera*, ultimately leaving us with 196 bacteria presenting in our initial analysis. The GWAS details are shown in Supplementary Table S2.

Hemorrhagic stroke (ICH and SAH) was defined as non-traumatic subarachnoid hemorrhage or intracerebral hemorrhage (Ohashi et al., 2023). Therefore, we obtained GWAS summary data for ICH, SAH, and nontraumatic intracranial hemorrhage (nITH) from the FinnGen Consortium’s R9 data. Specifically, the dataset for ICH consisted of 3,749 cases and 339,914 controls; the dataset for SAH included 3,289 cases and 339,922 controls; and the dataset for nITH included 6,530 cases and 342,673 controls. All the populations included in these datasets belonged to European groups (details shown in Supplementary Table S2).

The GWAS summary data for the risk factors of hemorrhagic stroke, including hypertension, obesity, alcohol drinking, and diabetes, were obtained from the corresponding consortia of the Integrative Epidemiology Unit (IEU) Open GWAS Project (Supplementary Table S2).

### 2.3 Selection of instrumental variable

We used SNPs with *p* < 1 × 10^−5^ as the initial genetic instrumental variable for MR analyses. This value was determined to be the optimal threshold for many intestinal flora-associated MR studies to maximize the amount of genetic variance explained by the genetic predictors and to increase the number of eligible SNPs for sensitivity analyses (Sanna et al., 2019). Meanwhile, the *F*-statistic serves as a reliable measure to evaluate the robustness of instruments. It is commonly computed using the formula:

$$F=\frac{R^{2}\left(n-1-k\right)}{\left(1-R^{2}\right)k}$$

with *R*^2^ indicating the degree of variance elucidated by the instruments, *n* signifying the sample size, and *k* representing the number of chosen IVs. To prevent potential weak instrument bias in our research, we have established a conventional threshold value of *F*-statistic >10; SNPs with *F*-statistic less than 10 were excluded (Staiger and Stock, 1994). To prevent the influence of linkage disequilibrium (LD) on the findings, the included genetic variants were screened using the clumping approach with *r*^2^ > 0.001 and clump window <10,000 kb (Li et al., 2022). we then removed the SNP that was significantly associated with hemorrhagic stroke based on the following criterion: *P*_outcome_ < *P*_exposure_. Finally, we removed SNPs with palindromic structures to ensure the screened SNPs aligned at the exposure and outcome alleles.

### 2.4 Statistical analysis of mendelian randomization

For Univariable MR analysis (UVMR), we used a variety of MR-related statistical methods, including inverse variance weighted (IVW), MR-Egger regression, weighted median, weighted mode, simple mode, and MR-PRESSO to examine whether there is a causal relationship between GM and hemorrhagic stroke. The IVW method allows us to estimate the causal effect of exposure on the outcome by incorporating ratio estimates for each SNP (Choi et al., 2019). Essentially, it transforms MR estimates into a weighted regression of SNP-outcome effects on SNP-exposure effects. While the weighted median method can produce unbiased estimates even when up to 50% of the data derives from invalid IVs (Bowden et al., 2016), the weighted mode approach is reliable when most individual instrument causal effect estimates are derived from valid instruments (Hartwig et al., 2017), even if some IVs are considered invalid. The MR-Egger method is a valuable tool for estimating causal effects through the slope coefficient from Egger regression, which helps identify and address potential small study bias (Bowden et al., 2015). Additionally, the simple mode represents an unweighted empirical density function for causal estimation (Hemani et al., 2018). The IVW provides the most precise estimates among these methods, assuming that all SNPs are valid instruments. If the IVW method yields a significant result (*p* < 0.05), even when other methods do not, and there is no evidence of pleiotropy or heterogeneity, the outcome can be considered positive as long as the beta values of the other methods point in the same direction (Chen et al., 2020). Meanwhile, the *p*-value of MR-PRESSO was less than 0.05, reinforcing the robustness of the positive results.

For the primary MR results, we corrected the *p*-values for False discovery rate (FDR) based on different taxa of GM and type of hemorrhagic stroke, resulting in corresponding *P*_FDR_ value with a false discovery rate of *P*_FDR_ < 0.1 (Storey and Tibshirani, 2003). A potential significant causality between the exposure and the outcome was considered to be present when *p* < 0.05 but *P*_FDR_ ≥ 0.1.

For both significant and potential significant causalities, we employed MR-Egger regression to assess whether the genetic instruments had a pleiotropic effect on the outcome (Bowden et al., 2015). Additionally, MR-PRESSO was utilized to identify horizontal pleiotropy and heterogeneity and mitigate its effect by removing outliers. The Cochran’s *Q* test was also applied to detect heterogeneity. If heterogeneity was detected by Cochran’s *Q* test, along with horizontal pleiotropy of selected SNPs by MR-Egger (egger-intercept) or MR-PRESSO (Global test), the analysis was repeated after excluding these pleiotropic SNPs. An insignificant *p*-value (*p* > 0.05) indicated the absence of heterogeneity or pleiotropy. Additionally, to ensure unbiased causal estimates, we performed leave-one-out analyses by iteratively excluding each instrumental SNP and re-running the IVW analyses, enabling the identification of any influential SNP (Li et al., 2022).

In order to assess the complete correlation between GM and hemorrhagic stroke, we conducted a reverse MR analysis using GM, which had been previously identified by forward MR analysis to have a causal relationship with hemorrhagic stroke. The settings and methodologies employed were consistent with those used in the forward MR analysis. In addition, some confounding factors (hypertension, obesity, alcohol drinking, and diabetes) may influence the causal relationship between GM and hemorrhagic stroke. In order to fully adjust for confounding factors, we included the above confounders (hypertension, obesity class 3, alcohol consumption, and type 2 diabetes) further using multivariate MR analysis (MVMR) in the forward MR analysis. Similarly, IVW, weighted median, and MR-Egger regression were used for MVMR analysis. The Egger-intercept test and Cochran’s *Q* test were also used to assess the stability of the results.

All statistical analyses were conducted with R (version 4.3.1), and MR analyses were executed using the TwosampleMR (Hemani et al., 2018), MR-PRESSO (Verbanck et al., 2018), and Mendelian Randomization R packages.

## 3 Results

### 3.1 Selection of instrumental variables

Strictly based on the selection criteria of IVs, we excluded four intestinal bacterial taxa that did not make it into the MR analysis. Finally, 192 intestinal bacterial taxa were included in the MR analysis. In total, we performed MR analysis after selecting SNPs that met the criteria for ICH (2030), SAH (2030) and nITH (2032).

### 3.2 Causal effects of gut microbiota on hemorrhagic stroke by UVMR

**Figure 2:**
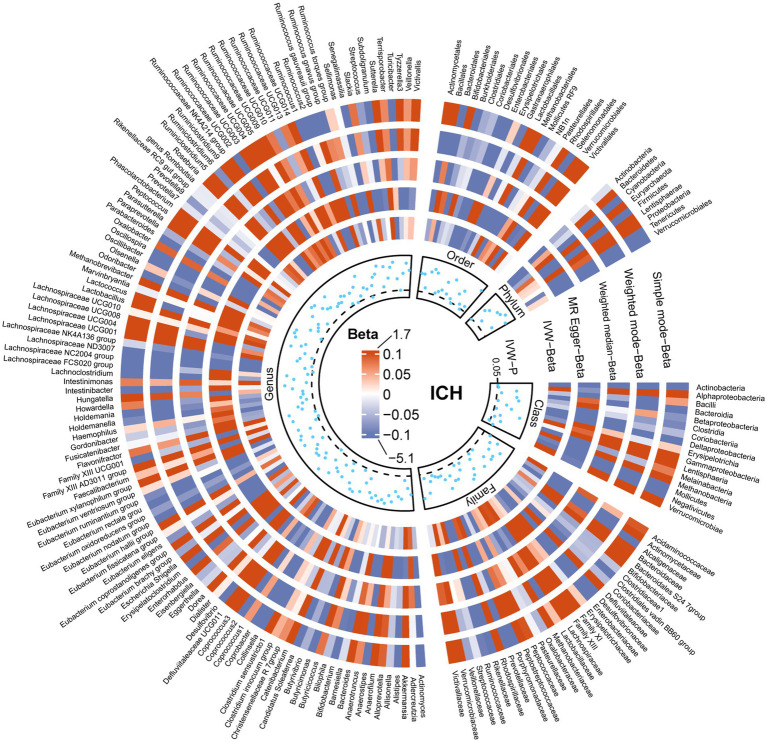
Causal effect of the gut microbiota on intracerebral hemorrhage based on MR analyses. From outside to inside, the beta values of simple mode, weighted mode, weighted median, MR-Egger, inverse variance weighted, and *p*-value inverse variance weighted are represented, respectively. MR, Mendelian randomization; IVW, Inverse variance weighted.

**Figure 3:**
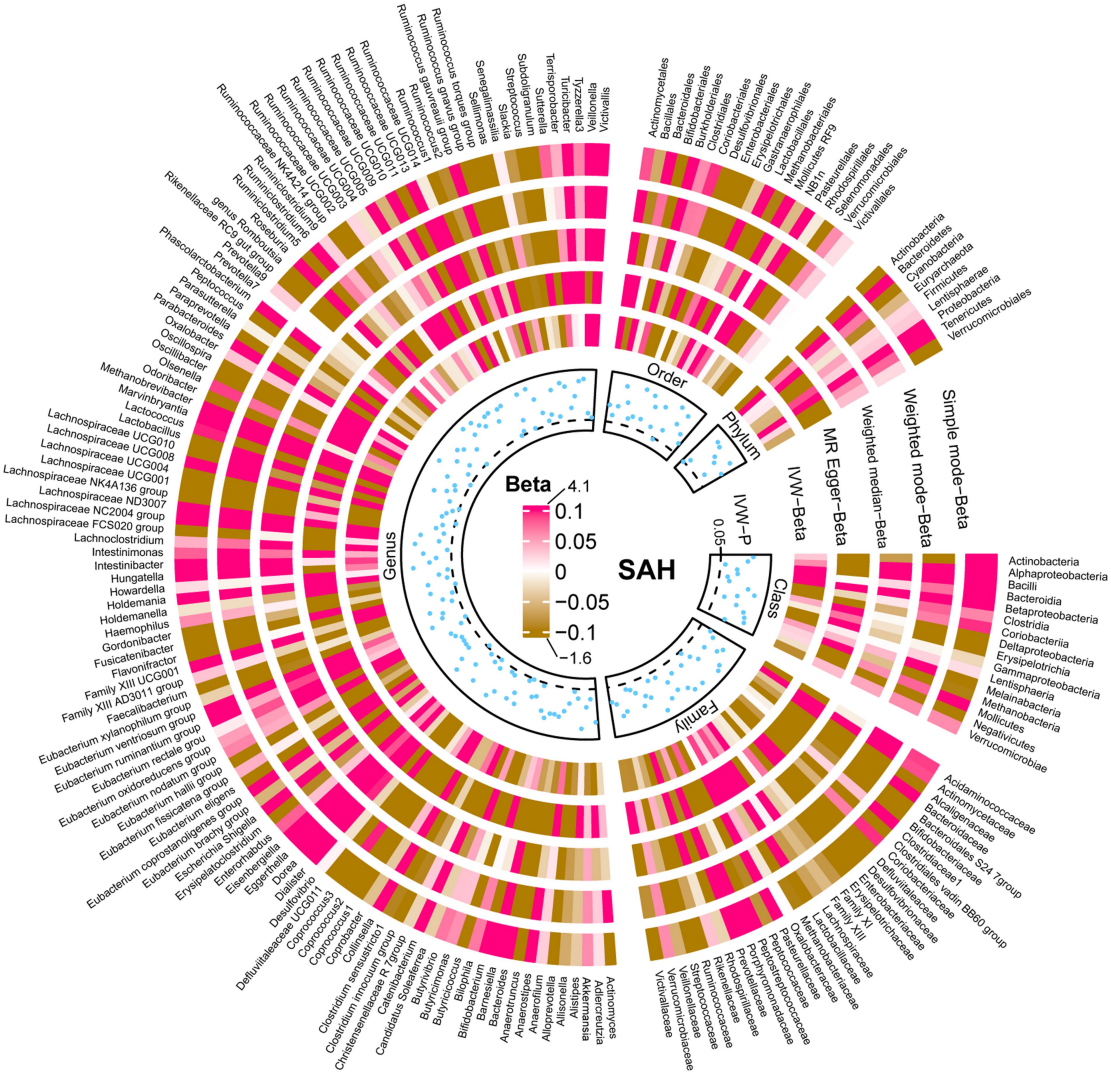
Causal effect of the gut microbiota on subarachnoid hemorrhage based on MR analyses. From outside to inside, the beta values of simple mode, weighted mode, weighted median, MR-Egger, inverse variance weighted, and *p*-value inverse variance weighted are represented, respectively. MR, Mendelian randomization; IVW, Inverse variance weighted.

**Figure 4:**
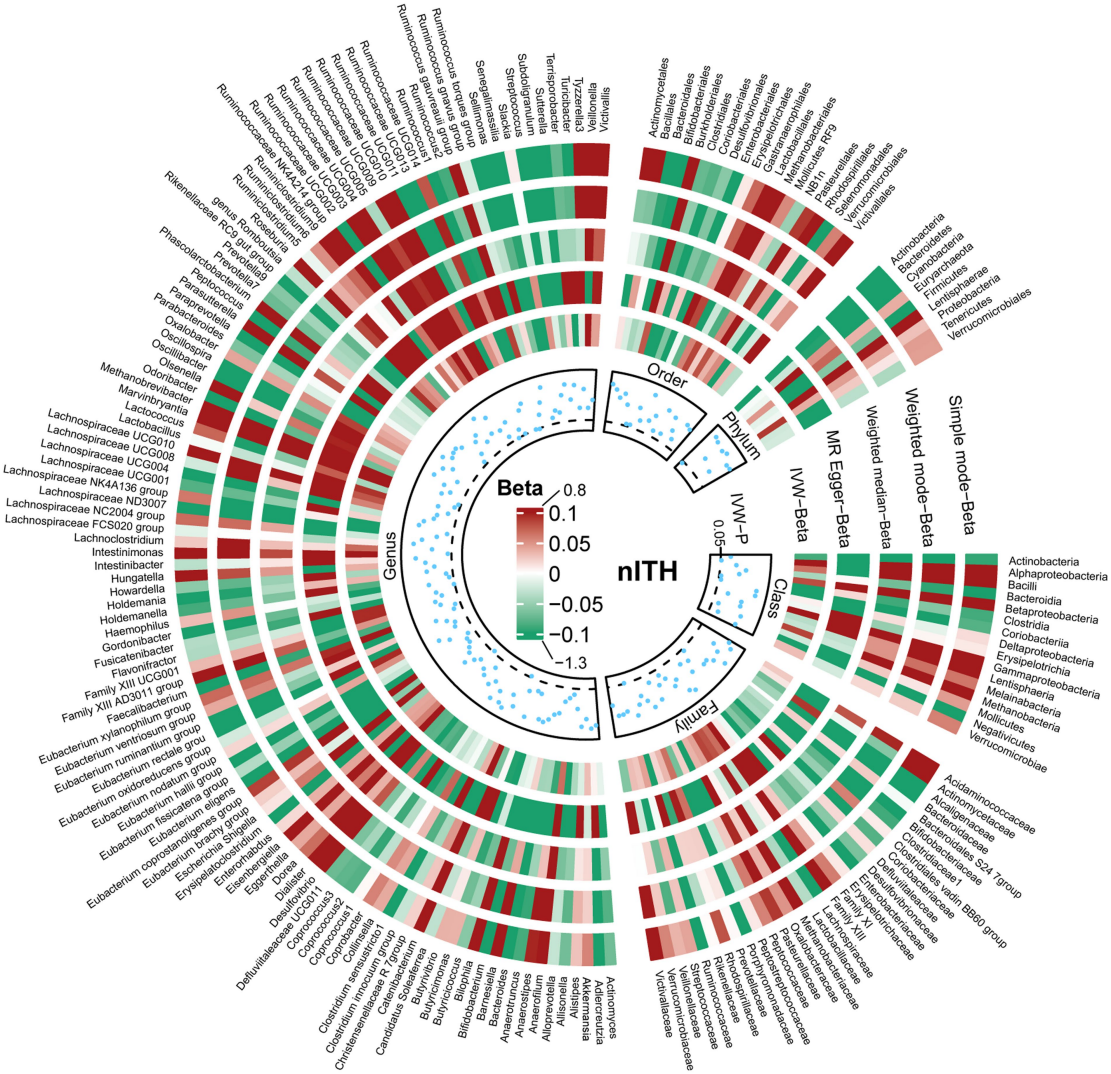
Causal effect of the gut microbiota on nontraumatic intracranial hemorrhage based on MR analyses. From outside to inside, the beta values of simple mode, weighted mode, weighted median, MR-Egger, inverse variance weighted, and *p*-value inverse variance weighted are represented, respectively. MR, Mendelian randomization; IVW, Inverse variance weighted.

**Figure 5:**
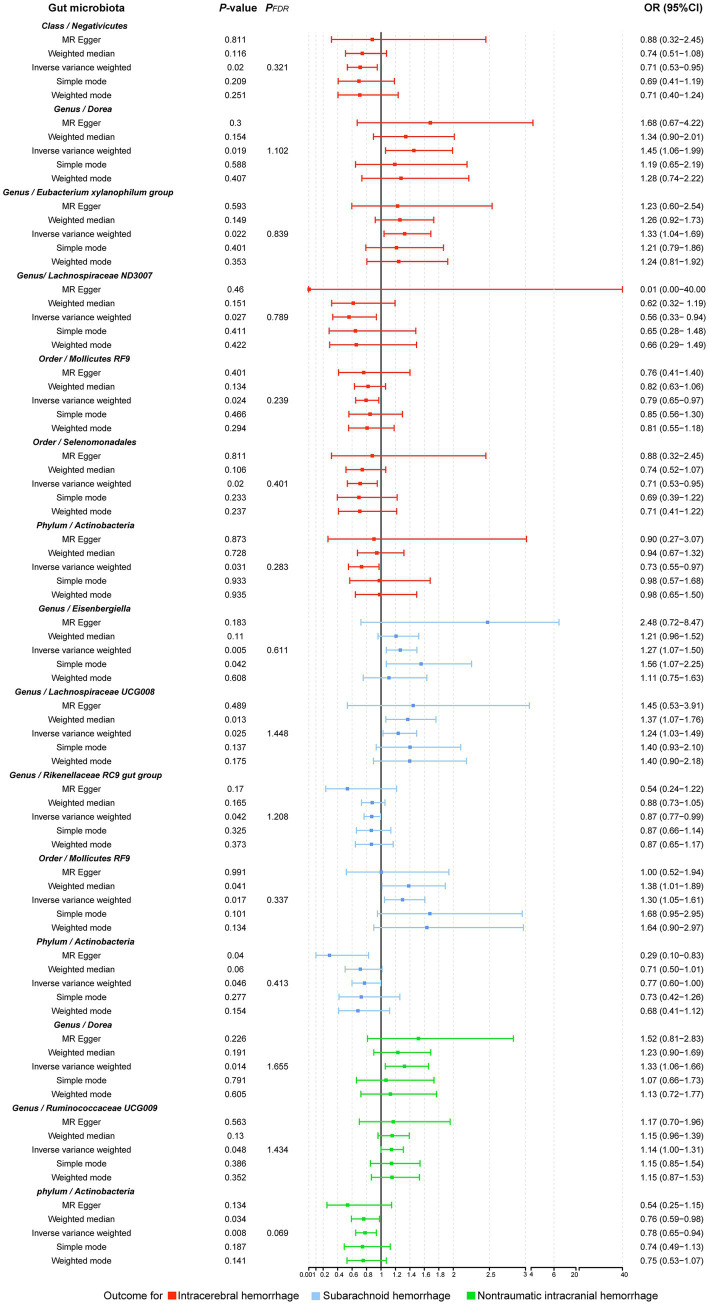
Forest plot illustrating the causal effect of the gut microbiota on hemorrhagic stroke using five methods of MR analyses.

**Figure 6:**
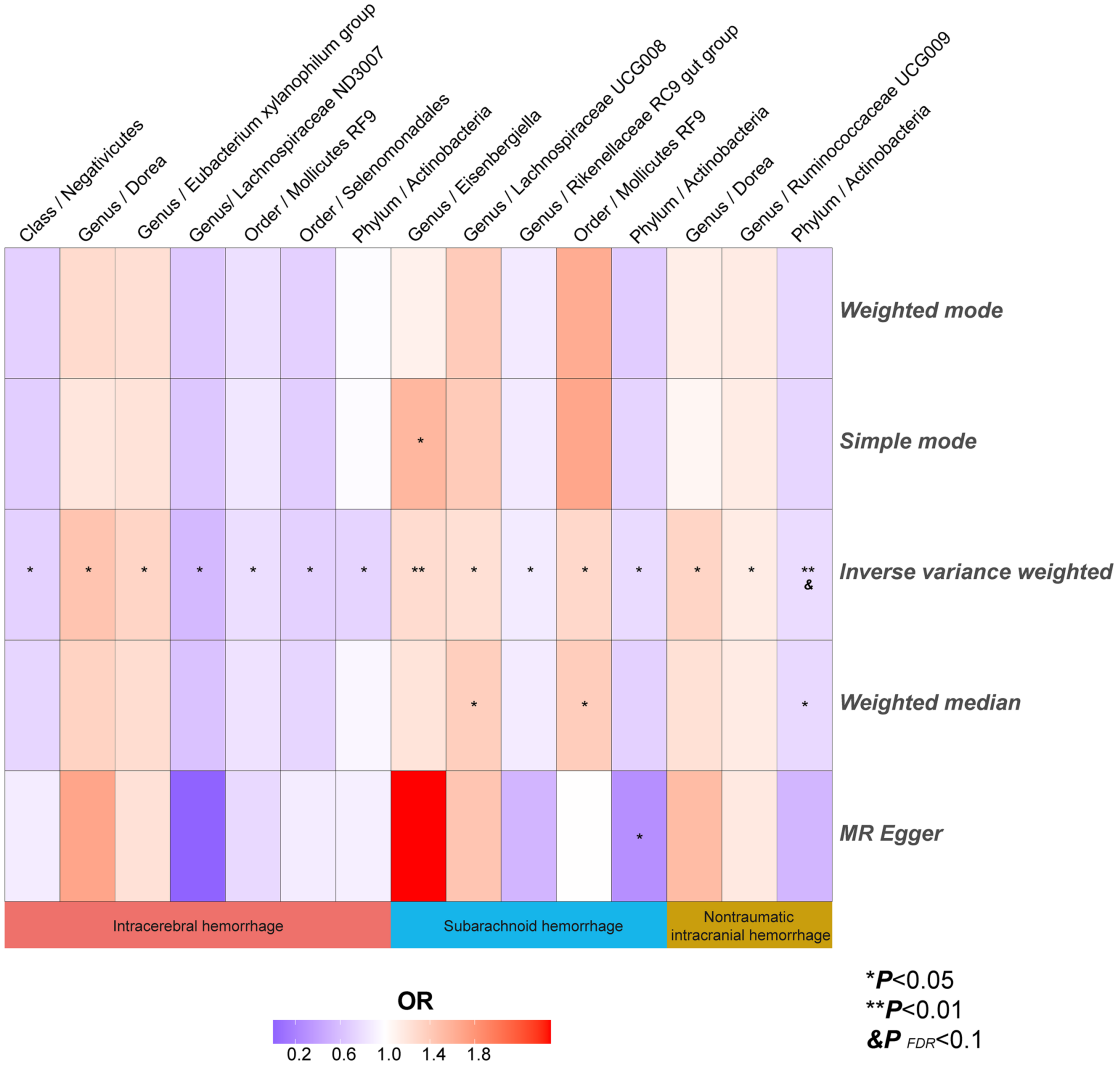
The heatmaps of five MR analysis methods. Different color blocks represent different odds ratio values. MR, Mendelian randomization.

The results of all MR analyses are shown in Figures 2–4, which record the beta values of all MR analysis methods and the *p*-values of the IVW method. And then, we identified the positive results of the MR analysis based on IVW’s significant *p*-values. In addition, the direction of the beta results for all five methods is consistent, increasing the credibility of the true causal relationships (Table 1, Figures 5, 6). The *F*-statistics of the IVs included in the analysis significantly associated with the GM were all greater than 10, indicating that the estimates are unlikely to suffer from weak instrumental bias (Supplementary Table S1).

**Table 1:** Mendelian randomization analysis results between Gut microbiota and hemorrhagic stroke.

Type of hemorrhagic stroke/outcome	Taxa	Gut microbiota	nSNP	Method of MR	SE	*p*-value	OR (95%CI)	*p*_FDR_
Intracerebral hemorrhage	Class	Negativicutes	12	MR Egger	0.523	0.811	0.88 (0.32–2.45)	
				Weighted median	0.191	0.116	0.74 (0.51–1.08)	
				Inverse variance weighted	0.146	**0.020**	0.71 (0.53–0.95)	0.321
				Simple mode	0.273	0.209	0.69 (0.41–1.19)	
				Weighted mode	0.286	0.251	0.71 (0.40–1.24)	
	Genus	Dorea	10	MR Egger	0.469	0.300	1.68 (0.67–4.22)	
				Weighted median	0.207	0.154	1.34 (0.90–2.01)	
				Inverse variance weighted	0.160	**0.019**	1.45 (1.06–1.99)	1.102
				Simple mode	0.311	0.588	1.19 (0.65–2.19)	
				Weighted mode	0.281	0.407	1.28 (0.74–2.22)	
	Genus	*Eubacterium xylanophilum* group	9	MR Egger	0.369	0.593	1.23 (0.60–2.54)	
				Weighted median	0.161	0.149	1.26 (0.92–1.73)	
				Inverse variance weighted	0.123	**0.022**	1.33 (1.04–1.69)	0.839
				Simple mode	0.219	0.401	1.21 (0.79–1.86)	
				Weighted mode	0.222	0.353	1.24 (0.81–1.92)	
	Genus	Lachnospiraceae ND3007	3	MR Egger	4.465	0.460	0.01 (0.00–40.00)	
				Weighted median	0.338	0.151	0.62 (0.32–1.19)	
				Inverse variance weighted	0.264	**0.027**	0.56 (0.33–0.94)	0.789
				Simple mode	0.423	0.411	0.65 (0.28–1.48)	
				Weighted mode	0.418	0.422	0.66 (0.29–1.49)	
	Order	Mollicutes RF9	12	MR Egger	0.312	0.401	0.76 (0.41–1.40)	
				Weighted median	0.132	0.134	0.82 (0.63–1.06)	
				Inverse variance weighted	0.102	**0.024**	0.79 (0.65–0.97)	0.239
				Simple mode	0.216	0.466	0.85 (0.56–1.30)	
				Weighted mode	0.194	0.294	0.81 (0.55–1.18)	
	Order	Selenomonadales	12	MR Egger	0.523	0.811	0.88 (0.32–2.45)	
				Weighted median	0.186	0.106	0.74 (0.52–1.07)	
				Inverse variance weighted	0.146	**0.020**	0.71 (0.53–0.95)	0.401
				Simple mode	0.288	0.233	0.69 (0.39–1.22)	
				Weighted mode	0.277	0.237	0.71 (0.41–1.22)	
	Phylum	Actinobacteria	14	MR Egger	0.624	0.873	0.90 (0.27–3.07)	
				Weighted median	0.171	0.728	0.94 (0.67–1.32)	
				Inverse variance weighted	0.145	**0.031**	0.73 (0.55–0.97)	0.283
				Simple mode	0.277	0.933	0.98 (0.57–1.68)	
				Weighted mode	0.214	0.935	0.98 (0.65–1.50)	
Subarachnoid hemorrhage	Genus	Eisenbergiella	11	MR Egger	0.628	0.183	2.48 (0.72–8.47)	
				Weighted median	0.118	0.110	1.21 (0.96–1.52)	
				Inverse variance weighted	0.085	**0.005**	1.27 (1.07–1.50)	0.611
				Simple mode	0.189	0.042	1.56 (1.07–2.25)	
				Weighted mode	0.197	0.608	1.11 (0.75–1.63)		
	Genus	Lachnospiraceae UCG008	10	MR Egger	0.508	0.489	1.45 (0.53–3.91)	
				Weighted median	0.128	0.013	1.37 (1.07–1.76)	
				Inverse variance weighted	0.095	**0.025**	1.24 (1.03–1.49)	1.448
				Simple mode	0.207	0.137	1.40 (0.93–2.10)	
				Weighted mode	0.227	0.175	1.40 (0.90–2.18)	
	Genus	Rikenellaceae RC9 gut group	11	MR Egger	0.418	0.170	0.54 (0.24–1.22)	
				Weighted median	0.093	0.165	0.88 (0.73–1.05)	
				Inverse variance weighted	0.067	**0.042**	0.87 (0.77–0.99)	1.208
				Simple mode	0.139	0.325	0.87 (0.66–1.14)	
				Weighted mode	0.151	0.373	0.87 (0.65–1.17)	
	Order	Mollicutes RF9	12	MR Egger	0.336	0.991	1.00 (0.52–1.94)	
				Weighted median	0.159	0.041	1.38 (1.01–1.89)	
				Inverse variance weighted	0.109	**0.017**	1.30 (1.05–1.61)	0.337
				Simple mode	0.289	0.101	1.68 (0.95–2.95)	
				Weighted mode	0.304	0.134	1.64 (0.90–2.97)	
	Phylum	Actinobacteria	14	MR Egger	0.541	0.040	0.29 (0.10–0.83)	
				Weighted median	0.179	0.060	0.71 (0.50–1.01)	
				Inverse variance weighted	0.130	**0.046**	0.77 (0.60–1.00)	0.413
				Simple mode	0.281	0.277	0.73 (0.42–1.26)	
				Weighted mode	0.254	0.154	0.68 (0.41–1.12)	
Nontraumatic intracranial hemorrhage	Genus	Dorea	10	MR Egger	0.318	0.226	1.52 (0.81–2.83)	
				Weighted median	0.160	0.191	1.23 (0.90–1.69)	
				Inverse variance weighted	0.115	**0.014**	1.33 (1.06–1.66)	1.655
				Simple mode	0.246	0.791	1.07 (0.66–1.73)	
				Weighted mode	0.229	0.605	1.13 (0.72–1.77)	
	Genus	Ruminococcaceae UCG009	11	MR Egger	0.262	0.563	1.17 (0.70–1.96)	
				Weighted median	0.095	0.130	1.15 (0.96–1.39)	
				Inverse variance weighted	0.068	**0.048**	1.14 (1.00–1.31)	1.434
				Simple mode	0.150	0.386	1.15 (0.85–1.54)	
				Weighted mode	0.145	0.352	1.15 (0.87–1.53)	
	Phylum	Actinobacteria	14	MR Egger	0.387	0.134	0.54 (0.25–1.15)	
				Weighted median	0.129	0.034	0.76 (0.59–0.98)	
				Inverse variance weighted	0.093	**0.008**	0.78 (0.65–0.94)	**0.069**
				Simple mode	0.212	0.187	0.74 (0.49–1.13)	
				Weighted mode	0.180	0.141	0.75 (0.53–1.07)	

MR, Mendelian randomization; SE, Standard Error; OR, Odds ratio. The positive values are highlighted in bold.

#### 3.2.1 ICH

Analysis of the MR showed that the relative abundance of the genetically predicted 1 *class (Negativicutes)*, 3 *genera (Dorea, Eubacterium xylanophilum group, Lachnospiraceae ND3007),2 order (Mollicutes RF9, Selenomonadales)*, *1 phylum (Actinobacteria)* were potentially associated with the risk of ICH. Specifically, *Negativicutes (class)* (OR, 0.71; 95%CI, 0.53–0.95; *p* = 0.020), *Lachnospiraceae ND3007 (genus),* (OR, 0.56; 95%CI, 0.33–0.94; *p* = 0.027), *Mollicutes RF9 (order)* (OR, 0.79; 95%CI, 0.65–0.97; *p* = 0.024), *Selenomonadales (order)* (OR, 0.71; 95%CI, 0.53–0.95; *p* = 0.020), *Actinobacteria (phylum)* (OR, 0.73; 95%CI, 0.55–0.97; *p* = 0.031) were potentially protective against ICH, i.e., higher abundance of them, the lower the risk of ICH may be. The relative abundance of *Dorea (genus)* (OR, 1.45; 95%CI, 1.06–1.99; *p* = 0.019) and *Eubacterium xylanophilum group (genus)* (OR, 1.33; 95%CI, 1.04–1.69; *p* = 0.022) may contribute to the development of ICH, and the higher the abundance of these two genera, the higher the potential risk of ICH occurrence (Table 1, Figures 5, 6).

#### 3.2.2 SAH

Similarly, the relative abundance of genetically predicted taxa showed potential significant associations with the risk of developing SAH. Specifically, the *Eisenbergiella (genus)* (OR, 1.27; 95%CI, 1.07–1.50; *p* = 0.005), *Lachnospiraceae UCG008 (genus)* (OR, 1.24; 95%CI, 1.03–1.49; *p* = 0.025) and *Mollicutes RF9 (order)* (OR, 1.30; 95%CI, 1.05–1.61; *p* = 0.017) were potential positive association with SAH, with their higher relative abundance associated with a higher potential risk of disease. Conversely, for the *Rikenellaceae RC9 gut group (genus)* (OR, 0.87; 95%CI, 0.77–0.99; *p* = 0.042) and *Actinobacteria (phylum)* (OR, 0.77; 95%CI, 0.60–1.00; *p* = 0.046), the higher the relative abundance, the lower the potential risk of developing SAH (Table 1, Figures 5, 6).

#### 3.2.3 nITH

In order to include the completeness of the population, we also selected the nITH population for MR analysis. The results showed that 2 *genera* and 1 *phylum* had a significant causal relationship with nITH. Specifically, *Actinobacteria (phylum)* (OR, 0.78; 95%CI, 0.65–0.94; *p* = 0.008, *P*_FDR_ = 0.069) was negatively associated with the risk of nITH. In other words, it was a protective factor against nITH. In contrast, *Dorea (genus)* (OR, 1.33; 95%CI, 1.06–1.66; *p* = 0.014), *Ruminococcaceae UCG009 (genus)* (OR, 1.14; 95%CI, 1.00–1.31; *p* = 0.048) were potentially positively associated with the risk of nITH (Table 1, Figures 5, 6).

### 3.3 Sensitivity analysis

In the sensitivity analyses, we performed pleiotropy, heterogeneity test, and leave-one-out analysis, respectively. All MR-Egger regression intercepts did not significantly deviate from zero (all intercepts *p* > 0.05). Cochran’s *Q* test and MR-PRESSO had *p*-values greater than 0.05; all of the above suggested that there was no significant heterogeneity and pleiotropy (shown in Supplementary Tables S4, S5). In addition, The leave-one-out analysis revealed no specific SNPs driving the association between GM and hemorrhagic stroke (Supplementary Table S6).

### 3.4 Causal effects of hemorrhagic stroke on gut microbiota

By reverse MR analysis, we found the abundance of *Dorea (genus)* (β, 0.05; 95%CI, 0.002 ~ 0.101; *p* = 0.041) may be up-regulated after ICH. Furthermore, the abundance of *Eisenbergiella (genus)* (β, −0.072; 95%CI, −0.137 ~ −0.007; *p* = 0.030, *P*_FDR_ = 0.090) was down-regulated when SAH acted as an exposure (shown in Supplementary Tables S7, S8). In the reverse MR analysis, we did not find heterogeneity and pleiotropy, and the leave-one-out analysis suggested that no SNP was significantly associated with the results (Supplementary Tables S9, S10).

### 3.5 Causal effects of exposure on hemorrhagic stroke by MVMR

**Figure 7:**
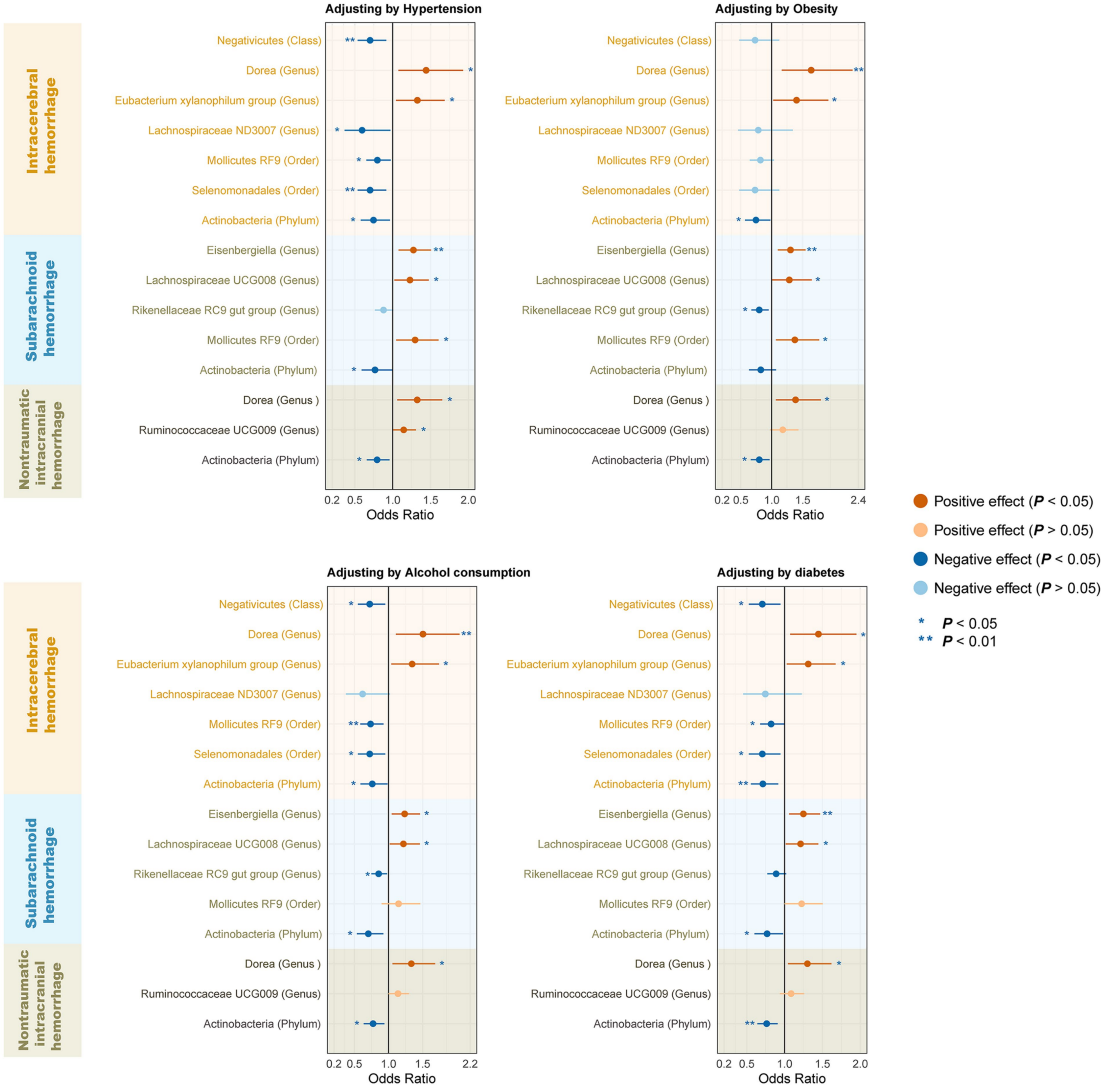
Forest plot illustrating the causal effect of gut microbiota on hemorrhagic stroke using the IVW method to adjust for each of the four confounders. IVW, Inverse variance weighted.

**Figure 8:**
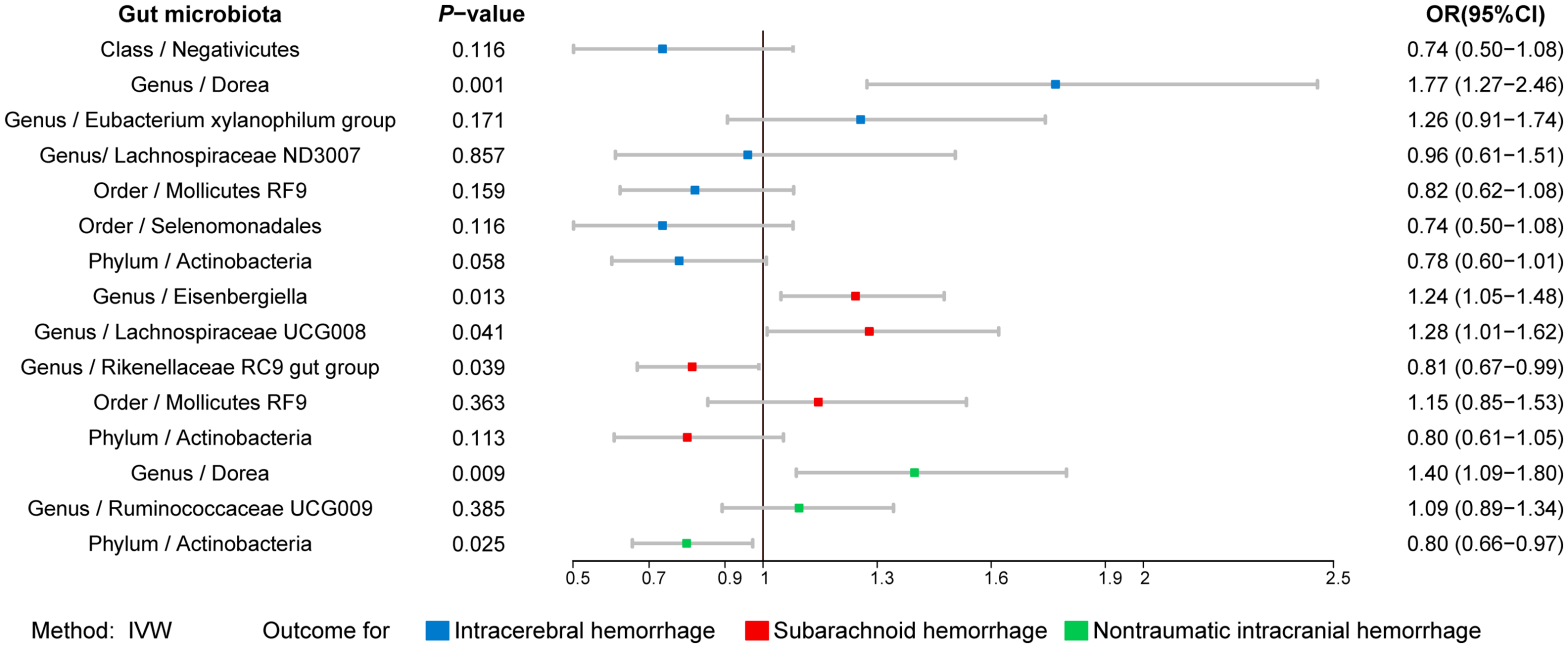
Forest plot illustrating the causal effect of gut microbiota on hemorrhagic stroke using IVW methods with simultaneous adjustment for four confounders. IVW, Inverse variance weighted.

To determine whether the above-selected microbiota exerted an impact on hemorrhagic stroke risk directly or through common hemorrhagic stroke confounders, we further conducted an MVMR analysis. In the MR analysis with different types of hemorrhagic stroke as the outcome, we considered confounding variables such as hypertension, obesity class 3, alcohol consumption, and type 2 diabetes separately, and the final results are shown in Figure 7 and Supplementary Table S11. After we subsequently considered all confounding variables simultaneously, the results are shown in Figure 8 and Table 2. Specifically, when ICH was the outcome, only *Dorea (genus)* remained as a potential risk factor for ICH. When SAH was used as the outcome, potential risk factors for SAH remained for *Eisenbergiella (genus)* and *Lachnospiraceae UCG008 (genus)*, and potential protective factors for SAH remained for *Rikenellaceae RC9 gut group (genus)*. When nITH was the outcome, potential risk factors for nITH were present for *Dorea (genus),* and protective factors for nITH were present for *Actinobacteria (phylum)*. The causal inference was further supported by consistent direction from distinct MR models. Besides, Cochran’s *Q* test and intercept term derived from multivariable IVW and multivariable MR-Egger did not detect potential heterogeneity and pleiotropy (Supplementary Table S12).

**Table 2:** Multivariable mendelian randomization analysis results between gut microbiota and hemorrhagic stroke (adjusting four variables).

Type of hemorrhagic stroke/outcome	Taxa/gut microbiota	nSNP	Methods of multivariable MR	Beta	SE	*p*-value	OR (95%CI)
Intracerebral hemorrhage	Class/Negativicutes	36	Multivariable IVW	−0.307	0.195	0.116	0.74 (0.50–1.08)
	Class/Negativicutes		Multivariable Median	−0.390	0.289	0.177	0.68 (0.38–1.19)
	Class/Negativicutes		Multivariable Egger	−0.451	0.262	0.085	0.64 (0.38–1.06)
	Genus/Dorea	38	Multivariable IVW	0.570	0.168	**0.001**	**1.77 (1.27–2.46)**
	Genus/Dorea		Multivariable Median	0.705	0.239	**0.003**	2.02 (1.27–3.23)
	Genus/Dorea		Multivariable Egger	0.472	0.212	**0.026**	1.60 (1.06–2.43)
	Genus/*Eubacterium xylanophilum* group	36	Multivariable IVW	0.228	0.167	0.171	1.26 (0.91–1.74)
	Genus/*Eubacterium xylanophilum* group		Multivariable Median	0.067	0.231	0.771	1.07 (0.68–1.68)
	Genus/*Eubacterium xylanophilum* group		Multivariable Egger	0.253	0.199	0.204	1.29 (0.87–1.90)
	Genus/ Lachnospiraceae ND3007	34	Multivariable IVW	−0.041	0.230	0.857	0.96 (0.61–1.51)
	Genus/ Lachnospiraceae ND3007		Multivariable Median	−0.245	0.309	0.427	0.78 (0.43–1.43)
	Genus/ Lachnospiraceae ND3007		Multivariable Egger	−0.237	0.337	0.482	0.79 (0.41–1.53)
	Order/Mollicutes RF9	38	Multivariable IVW	−0.197	0.140	0.159	0.82 (0.62–1.08)
	Order/Mollicutes RF9		Multivariable Median	−0.119	0.200	0.550	0.89 (0.60–1.31)
	Order/Mollicutes RF9		Multivariable Egger	−0.414	0.196	**0.035**	0.66 (0.45–0.97)
	Order/Selenomonadales	36	Multivariable IVW	−0.307	0.195	0.116	0.74 (0.50–1.08)
	Order/Selenomonadales		Multivariable Median	−0.390	0.289	0.177	0.68 (0.38–1.19)
	Order/Selenomonadales		Multivariable Egger	−0.451	0.262	0.085	0.64 (0.38–1.06)
	Phylum/Actinobacteria	42	Multivariable IVW	−0.249	0.132	0.058	0.78 (0.60–1.01)
	Phylum/Actinobacteria		Multivariable Median	−0.026	0.183	0.888	0.97 (0.68–1.39)
	Phylum/Actinobacteria		Multivariable Egger	0.045	0.200	0.823	1.05 (0.71–1.55)
Subarachnoid hemorrhage	Genus/Eisenbergiella	42	Multivariable IVW	0.218	0.088	**0.013**	**1.24 (1.05–1.48)**
	Genus/Eisenbergiella		Multivariable Median	0.261	0.119	**0.028**	1.30 (1.03–1.64)
	Genus/Eisenbergiella		Multivariable Egger	0.287	0.115	**0.012**	1.33 (1.06–1.67)
	Genus/Lachnospiraceae UCG008	37	Multivariable IVW	0.246	0.120	**0.041**	**1.28 (1.01–1.62)**
	Genus/Lachnospiraceae UCG008		Multivariable Median	0.302	0.171	0.078	1.35 (0.97–1.89)
	Genus/Lachnospiraceae UCG008		Multivariable Egger	0.203	0.162	0.209	1.23 (0.89–1.68)
	Genus/Rikenellaceae RC9 gut group	35	Multivariable IVW	−0.206	0.100	**0.039**	**0.81 (0.67–0.99)**
	Genus/Rikenellaceae RC9 gut group		Multivariable Median	−0.106	0.132	0.422	0.90 (0.69–1.16)
	Genus/Rikenellaceae RC9 gut group		Multivariable Egger	−0.248	0.125	**0.048**	0.78 (0.61–1.00)
	Order/Mollicutes RF9	38	Multivariable IVW	0.136	0.149	0.363	1.15 (0.85–1.53)
	Order/Mollicutes RF9		Multivariable Median	0.087	0.206	0.672	1.09 (0.73–1.63)
	Order/Mollicutes RF9		Multivariable Egger	0.275	0.209	0.187	1.32 (0.87–1.98)
	Phylum/Actinobacteria	42	Multivariable IVW	−0.222	0.140	0.113	0.80 (0.61–1.05)
	Phylum/Actinobacteria		Multivariable Median	−0.283	0.190	0.137	0.75 (0.52–1.09)
	Phylum/Actinobacteria		Multivariable Egger	−0.348	0.212	0.102	0.71 (0.47–1.07)
nontraumatic intracranial hemorrhage	Genus/Dorea	41	Multivariable IVW	0.335	0.128	**0.009**	**1.40 (1.09–1.80)**
	Genus/Dorea		Multivariable Median	0.421	0.187	**0.024**	1.52 (1.06–2.20)
	Genus/Dorea		Multivariable Egger	0.274	0.162	0.090	1.32 (0.96–1.81)
	Genus/Ruminococcaceae UCG009	36	Multivariable IVW	0.091	0.104	0.385	1.09 (0.89–1.34)
	Genus/Ruminococcaceae UCG009		Multivariable Median	0.096	0.130	0.458	1.10 (0.85–1.42)
	Genus/Ruminococcaceae UCG009		Multivariable Egger	0.197	0.140	0.160	1.22 (0.93–1.60)
	Phylum/Actinobacteria	42	Multivariable IVW	−0.225	0.100	**0.025**	**0.80 (0.66–0.97)**
	Phylum/Actinobacteria		Multivariable Median	−0.241	0.136	0.077	0.79 (0.60–1.03)
	Phylum/Actinobacteria		Multivariable Egger	−0.075	0.152	0.624	0.93 (0.69–1.25)

Four confounding variables were adjusted simultaneously, including hypertension, obesity class 3, alcohol consumption, and type 2 diabetes. MR, Mendelian randomization; IVW, Inverse variance weighted. The positive values are highlighted in bold.

## 4 Discussion

To our best knowledge, this is the first MR analysis of GM for hemorrhagic stroke. We conducted bidirectional, two-sample, and MVMR analysis to assess the causal relationship between GM and hemorrhagic stroke. Our findings revealed that after adjusting for a range of confounders, *Actinobacteria (phylum)* had a protective effect against hemorrhagic stroke, while *Rikenellaceae RC9 gut group (genus)* had a potential protective effect. On the other hand, *Dorea (genus)*, *Eisenbergiella (genus),* and *Lachnospiraceae UCG008 (genus)* acted as potential risk factors for hemorrhagic stroke. The occurrence of hemorrhagic stroke also alters the composition of the GM, as evidenced primarily by a potential increase in the relative abundance of *Dorea (genus)* and a decrease in the relative abundance of *Eisenbergiella (genus)*. These findings suggest the potential value of GM in the identification, prevention, and management of hemorrhagic stroke.

GM plays a crucial role in human growth, development, immune responses, metabolism, and various pathophysiological processes. It exerts a direct or indirect influence on numerous aspects, including host cell proliferation, angiogenesis, intestinal endocrine function, nerve signaling, and the synthesis of essential substances (Kåhrström et al., 2016; Fan and Pedersen, 2021). Moreover, GM affects remote organs, such as the liver, kidneys, and even the central nervous system. MGB axis introduction leads to a better understanding of the onset and progression of neurological disorders (Mulak and Bonaz, 2015).

Drawing from the MGB axis, connections have been identified linking GM to an array of neurological disorders, including Parkinson’s disease, Alzheimer’s disease, and ischemic stroke. After the commencement of ischemic stroke, ischemia induces gastrointestinal paralysis and an excessive generation of nitrates, leading to a modified flora composition (Xu et al., 2021). The *Aerococcaceae (family)* and *Flavobacterium (genus)* demonstrated a substantial increase. Conversely, the *Clostridiales (order)*, as well as the *genera Lactobacillus* and *Stenotrophomonas*, exhibited a marked decrease within the intestinal tracts of ischemic stroke patients when contrasted with the control group. Furthermore, the flora’s composition proved capable of effectively discerning between patients with differing prognoses (Chang et al., 2021). Additionally, a diminished presence of GM responsible for butyrate production is an autonomous predictor for post-stroke infection development (Haak et al., 2021). Substantial strides have been achieved in recent years concerning alterations in the abundance and composition of GM within patients afflicted by ICH. In a cohort study investigating GM in ICH patients, significant decreases in the abundance of the *genera Brucella*, *Bacteroidota*, *Fusobacterium*, *E. faecalis*, *Bifidobacterium*, *Romboutsia*, and *Agathobacteria* were observed. Conversely, significant increases in the abundance of the *genera Ackermannia*, *Escherichia*, *Shigella*, *Clostridium*, and *Lactobacillus* were observed after the onset of ICH (Xiong et al., 2022). A separate study identified increased levels of *Enterococcus*, *Parabacteroides*, *Lachnoclostridium*, *Acidaminococcus*, and *Streptococcus* after ICH onset, accompanied by a notable reduction in the abundance of *Prevotella* and *Faecalibacterium* (Luo et al., 2022). Therefore, changes in the GM following hemorrhagic stroke have been continuously validated. Within our investigation, we examined the causal impact of hemorrhagic stroke on select members of the GM through reversed MR. Additionally, we supplemented our analysis with *Dorea (genus)* and *Eisenbergiella (genus)*, taxa that exhibited changes in response to hemorrhagic stroke.

The disruption of GM and metabolite composition further amplifies secondary damage after hemorrhagic stroke. Within preclinical investigations, mice within the ICH model displayed disrupted GM alongside an inflammatory reaction in the hematoma’s periphery, coupled with the presence of intestinal lymphocytes. Notably, mice that received a transplantation of regular GM exhibited a marked reduction in neuroinflammation (Yu et al., 2021). To put it differently, the modified GM following cerebral hemorrhage intensifies the harm caused by neuroinflammation—an element recognized as pivotal in secondary brain injury and unfavorable post-hemorrhagic stroke prognoses (Candelario-Jalil et al., 2022). Furthermore, ongoing inflammatory cell infiltration progressively harms vascular endothelial cells, resulting in heightened fragility of vessel walls along with both structural and functional decline. Consequently, this elevates the vulnerability to hemorrhagic stroke (Low et al., 2019). More specifically, in pathological conditions such as hemorrhagic stroke, the GM changes as described above. For example, there is a reduction in the populations of beneficial GM, while the presence of GM that produces harmful compounds increases. The subsequent gradual accumulation of harmful metabolites from GM induces changes in gut permeability. Histomorphological and ultrastructural examinations after ICH revealed deterioration of the intestinal epithelium. This was manifested as thickening, shortening, and fusion of the villi and fragmentation of epithelial cells on the surface of the villi (Cheng et al., 2016). Inflammation initiates a cascade of events resulting in harm to the intestinal epithelium, ongoing impairment of the intestinal barrier function, and eventual liberation of metabolites from intestinal bacteria into the circulatory system. Sequential stimulation of inflammatory pathway molecules in the bloodstream, including NOD-like receptor thermal protein domain associated protein-3 and Nuclear factor-κB, the inflammatory mediator Matrix metalloprotein-9, specific members of the interleukin family, and reactive oxygen species, induces the disruption of tight junctions within the blood–brain barrier’s architecture. Concurrently, vascular endothelial cell apoptosis occurs, further heightening susceptibility to hemorrhagic stroke and secondary cerebral edema (Guo et al., 2015; Zhang et al., 2022). Within a population-based study, an increase in *Enterococcus* abundance and a reduction in *Prevotella* abundance within the intestines of patients from the ICH group was observed to stimulate the progression of stroke-associated pneumonia, potentially via linked inflammatory factors (Luo et al., 2022), this outcome additionally bolsters the firmly interconnected and distinguishable correlation between GM and inflammation. *Dorea (genus)* is believed to be closely linked to inflammation and may worsen the severity of the disease when present in increased abundance, as seen in irritable bowel syndrome (Xu et al., 2021). Furthermore, the abundance of *Dorea (genus)* was significantly higher in an obese rat model, suggesting that it could contribute to the development of chronic inflammation in the organism (Jiao et al., 2018). In our study by MVMR analysis, *Dorea (genus)* was identified as a potential risk factor for stroke, suggesting that it may influence the development of hemorrhagic stroke through inflammation. *Eisenbergiella (genus)*, however, yielded inconsistent results. We found that *Eisenbergiella* could promote the development of hemorrhagic stroke, and it is believed that its abundance significantly rises in patients following a high-fat/low-fiber diet (Bailén et al., 2020). However, the abundance of *Eisenbergiella* was reduced after hemorrhagic stroke. We speculate that (1) differences may be due to population samples, and (2) *Eisenbergiella (genus)* can also contribute to the production of short-chain fatty acids (SCFAs) (Tian et al., 2022), which are thought to be closely associated with the development of secondary brain injury (Sadler et al., 2020). Thus, it may be low in abundance in the hemorrhagic stroke population.

We found that *Actinobacteria (phylum)* reduces the risk of hemorrhagic stroke. A current study found an elevated abundance of *Actinobacteria* in a rat model of hemorrhagic transformation compared to non-hemorrhagic transformation rats (Huang et al., 2022). Bifidobacterium belongs to *Actinobacteria (phylum)* and is thought to improve blood glucose and lipids, as well as act as an antioxidant (Tonucci et al., 2017). Meanwhile, *Actinobacteria (phylum)*, one of the major producers of antibiotics and butyrate, may reduce the risk of cerebrovascular lesions by controlling inflammation (Barka et al., 2016; Zhao et al., 2018). Butyrate is an essential SCFA that strengthens the intestinal barrier and exerts anti-inflammatory properties (Mills et al., 2019). Butyrate depletion is associated with risk factors for hemorrhagic stroke, such as diabetes, obesity, and neuroinflammation (Huang et al., 2022). Therefore, all of the factors mentioned above may help *Actinomycetes (phylum)* to reduce the risk of hemorrhagic stroke. It has also been shown that *the Rikenellaceae RC9 gut group (genus)* is significantly associated with resistance to oxidative stress and regulation of lipid metabolism, which explains its role as a protective bacterium against hemorrhagic stroke (Qin et al., 2022). On the other hand, *Lachnospiraceae UCG008 (genus)* is closely related to T cells and NK cells, suggesting that it may also have contributed to hemorrhagic stroke through an inflammatory response (Pang et al., 2022). Thus, inflammation-related factors may link GM and hemorrhagic stroke.

Given the complexities of conducting randomized controlled trials, we have used MR methods to delineate further the genetic-level determinants associated with hemorrhagic stroke, thereby improving the precision of early hemorrhagic stroke prevention and secondary injury mitigation. Nevertheless, large randomized controlled trials remain essential.

Our study has several limitations. First, the GWAS statistics are derived from a European population, which may limit the generalizability of the results. Second, due to the strict threshold filtering (*p* < 1 × 10^−5^) applied to IVs, there is a possibility of some potential spurious associations. Third, since we could not obtain individual-level data from the GWAS data, we were unable to analyze the detailed stratification of patient data, and thus were unable to explore the interactions between GM and different strata of hemorrhagic stroke patients. Despite this, as in this case, we were able to establish a causal relationship between GM and hemorrhagic stroke through a series of rigorous MR analyses, which set the foundation for our further studies.

## 5 Conclusion

We established a causal relationship between GM and the occurrence of hemorrhagic stroke through univariate and multivariate MR analyses. *Dorea (genus)*, *Eisenbergiella (genus)*, and *Lachnospiraceae UCG008 (genus)* may be associated with an increased risk of hemorrhagic stroke, whereas *Rikenellaceae RC9 gut group (genus)* and *Actinobacteria (phylum)* may be associated with a decreased risk of hemorrhagic stroke. Specifically, a higher abundance of *Actinobacteria (phylum)* is linked to a reduced risk of nITH. Inflammation likely plays a crucial role in connecting GM and hemorrhagic stroke. Future research should include more comprehensive investigations to verify the causal relationship between GM and hemorrhagic stroke, as well as to explore the underlying mechanisms.

## Data availability statement

Publicly available datasets were analyzed in this study. This data can be found here: the MiBioGen, https://mibiogen.gcc.rug.nl/menu/main/home; the FinnGen, https://r9.finngen.fi/; and the IEU Open GWAS, https://gwas.mrcieu.ac.uk/.

## Ethics statement

Ethical approval was not required for the studies involving humans because This study did not require additional ethical approval since we used publicly available data, which had already received approval from the appropriate ethical and institutional review boards. The studies were conducted in accordance with the local legislation and institutional requirements. Written informed consent for participation was not required from the participants or the participants’ legal guardians/next of kin in accordance with the national legislation and institutional requirements because this study did not require additional ethical approval since we used publicly available data, which had already received approval from the appropriate ethical and institutional review boards.

## Author contributions

YS: Conceptualization, Formal Analysis, Methodology, Visualization, Writing – original draft. HLiu: Data curation, Formal Analysis, Software, Writing – review & editing. XM: Methodology, Software, Validation, Writing – review & editing. AG: Investigation, Methodology, Supervision, Writing – review & editing. YL: Data curation, Investigation, Writing – review & editing. WM: Software, Visualization, Writing – review & editing. HLi: Conceptualization, Funding acquisition, Resources, Supervision, Writing – review & editing. FH: Conceptualization, Funding acquisition, Project administration, Resources, Writing – review & editing.
